# Elevated atmospheric CO_2_ delays the key timing for split N applications to improve wheat (*Triticum aestivum* L.) protein composition

**DOI:** 10.3389/fpls.2023.1186890

**Published:** 2023-06-20

**Authors:** Yue Pan, Xue Han, Huasen Xu, Wei Wu, Xiaoming Liu, Yingchun Li, Cheng Xue

**Affiliations:** ^1^ State Key Laboratory of North China Crop Improvement and Regulation/Key Laboratory for Farmland Eco-Environment of Hebei/College of Resources and Environmental Science, Hebei Agricultural University, Baoding, China; ^2^ Key Laboratory of Agro-environment and Climate Change of Agriculture Ministry, Institute of Environment and Sustainable Development in Agriculture, Chinese Academy of Agricultural Sciences, Beijing, China

**Keywords:** wheat, free-air CO_2_ enrichment (FACE), split N application, grain yield, protein

## Abstract

Late stage nitrogen (N) applications following basic fertilization are commonly used to ensure grain yield and increase grain protein content in wheat. Split N applications at the late growth stage of wheat are an effective measure to improve N absorption and transport and thus increase grain protein content. However, whether split N applications can alleviate the decrease in grain protein content induced by elevated atmospheric CO_2_ concentrations (e[CO_2_]) remains unclear. In the present study, a free-air CO_2_ enrichment system was used to investigate the effects of split N applications (at booting or anthesis) on grain yield, N utilization, protein content, and the composition of wheat under atmospheric (ACO_2_; 400 ± 15 ppm) and elevated CO_2_ concentrations (ECO_2_; 600 ± 15 ppm). The results showed that wheat grain yield and grain N uptake increased by 5.0% (being grains per ear by 3.0%, 1000-grain weight by 2.0%, and harvest index by 1.6%) and 4.3%, respectively, whereas grain protein content decreased by 2.3% under ECO_2_ conditions. Although the negative effect of e[CO_2_] on grain protein content was not alleviated by split N applications, gluten protein content was enhanced due to the alteration of N distribution in different protein fractions (albumins, globulins, gliadins, and glutenins). Compared to that without split N applications, the gluten content of wheat grains increased by 4.2% and 4.5% when late stage N was applied at the booting stage under ACO_2_ and anthesis under ECO_2_ conditions, respectively. The results indicate that rational handling of N fertilizers may be a promising approach to coordinating grain yield and quality under the effects of future climate change. However, compared to ACO_2_ conditions, the key timing for improving grain quality by split N applications should be postponed from the booting stage to anthesis under e[CO_2_] conditions.

## 1 Introduction

Elevated atmospheric CO_2_ concentrations (e[CO_2_]) are an important factor affecting global climate change. Atmospheric CO_2_ concentrations have increased by nearly 50% since the Industrial Revolution, owing to the massive combustion of fossil fuels and excessive deforestation (IPCC, 2022). It is estimated that atmospheric CO_2_ concentrations will increase from slightly over 410 ppm to 550 ppm by the mid-21st century and will further increase to 1000 ppm by the end of the 21st century if no effective restrictive measures are taken (Naidoo, 2022). As a substrate for photosynthesis, e[CO_2_] has an important effect on crop yield and quality.

As the second most stable crop in the world, wheat (*Triticum aestivum* L.) supplies approximately 20% and 20–40% of human nutritional protein and minerals, respectively, and occupies approximately 25% of the global cereal production area (Punia et al., 2017; FAO, 2019). Changes in atmospheric CO_2_ concentrations can affect the yield and quality of wheat by regulating its physiological and metabolic activities, thus influencing the development of roots, stems, leaves, and other organs (Wang et al., 2013; Dubey et al., 2015; Gojon et al., 2023). e[CO_2_] can increase wheat grain yield by promoting photosynthesis; however, the mechanism of the increase in yield components is inconsistent (Han et al., 2015; Jing et al., 2017). Some studies have found that e[CO_2_] increases wheat grain yield mainly by enhancing the ear number per unit area (Jing et al., 2017; Wang et al., 2023). However, other studies have indicated that an increase in the number of grains per spike is the main reason for the yield increase under e[CO_2_] (Kimball et al., 2010; Han et al., 2015). Nitrogen (N), an essential nutrient for plants, is mainly stored in wheat grains in the form of proteins, which can be further sequentially fractionated into albumin, globulin, gliadin, and glutenin proteins based on their solubility in different solvents (Rossmann et al., 2019; Peng et al., 2022). Gliadins and glutenins together form gluten proteins, which play a crucial role in influencing the processing quality of wheat flour. The processing quality of wheat flour is positively correlated with grain protein content (GPC) and gluten protein content (accounting for 50–80% of GPC) within a certain range (Xue et al., 2019). Unfortunately, the increase in grain yield affected by e[CO_2_] is often accompanied by a decrease in GPC, leading to a reduction in the processing quality of wheat flour (Bloom and Plant, 2021). Therefore, maintaining the increase in grain yield without reducing the processing quality is an urgent problem that requires investigation to ensure both grain yield and quality of wheat under future climate change conditions with respect to e[CO_2_].

Rational management of N fertilization is an effective measure to ensure both high yield and quality of wheat (Zörb et al., 2018). Many studies have been conducted on the influence of N fertilizer management on grain yield and quality of wheat under e[CO_2_] conditions (Dubey et al., 2015; Han et al., 2015; Dier et al., 2018; Pleijel et al., 2019; Bloom and Plant, 2021; Li et al., 2021). Unfortunately, the negative effect of e[CO_2_] on wheat GPC cannot be eliminated by simply increasing the N application rate (Pleijel et al., 2019). In addition, excessive N application leads to reduced N uptake and use efficiency due to N losses, thus increasing the risk of environmental pollution (Dier et al., 2019; Bloom and Plant, 2021). In addition to the N fertilization rate, the timing of N application also significantly affects wheat grain yield and quality (Blandino et al., 2015; Wu et al., 2022). The N source for protein synthesis in wheat grains is partly derived from the N stored in the vegetative organs before anthesis and then transported to the grains (accounting for approximately 60–95%) and partly from the N absorbed after anthesis and transported directly to the grains (accounting for approximately 5–40%); the latter is more conducive to grain protein synthesis (Blandino et al., 2015). Gluten proteins (gliadins and glutenins) are mainly synthesized and accumulated from approximately 7 (for gliadins) and 10 (for glutenins) days after anthesis until maturity (Gupta et al., 1996; Panozzo et al., 2001). Therefore, the application of N fertilizer during the late growth stages of wheat (e.g., at booting, anthesis, or even post-anthesis) is considered an effective way to achieve the desired GPC and thus improve the processing quality of wheat (Barneix, 2007). Besides, the interaction between various plant N statuses and hormones plays an important role in regulating the senescence of wheat leaves and the source-sink relationship of carbon and N (Abid et al., 2018). If a relatively high level of plant hormones (such as cytokinin and auxin) is maintained in wheat during the post-anthesis period due to a sufficient N supply, a higher storage capacity and filling capacity can be obtained, leading to increased wheat yield and N use efficiency (Zhang et al., 2021). Previous studies from our group have shown that the booting stage is the key timing for split N applications in late wheat to improve both grain yield and quality by altering N partitioning into different fractions of gluten proteins and their subunits in the wheat grain (Xue et al., 2016a; Xue et al., 2016b; Xue et al., 2019; Wu et al., 2022). e[CO_2_] could enhance the photosynthetic carbon assimilation of wheat, resulting in high N demand and promoting N uptake during vegetative growth and N translocation or distribution after wheat anthesis (Dier et al., 2019). However, no studies have reported whether the effects and mechanisms of late stage split N applications on the protein and quality of wheat still exist under e[CO_2_]. Therefore, further studies are needed to elucidate whether late stage split N applications could coordinate the correlation between grain yield and quality of wheat by alleviating the reduction in GPC as a result of the enhancement of grain yield under e[CO_2_]. In addition, the key timing for split N applications under e[CO_2_] could be later than that under atmospheric CO_2_ concentration conditions to increase GPC because the N absorbed post-anthesis is considered more conducive to grain protein synthesis (Blandino et al., 2015; Zörb et al., 2018).

Currently, the free-air CO_2_ enrichment (FACE) system is recognized as an ideal research method for studying the response of plants to e[CO_2_] conditions (Han et al., 2015; Li et al., 2021). This system is preferred over controllable chambers, open-top chambers, and other similar closed systems. In this study, the FACE system was used to investigate the responses of grain yield, protein and its composition, and N uptake and utilization of wheat, as affected by split N applications at different timings under e[CO_2_] conditions. The main objectives of this study were: (i) to determine whether split N applications at late growth stages of wheat could coordinate grain yield and quality of wheat under e[CO_2_]; (ii) to verify whether the key timing for split N applications should be postponed under e[CO_2_] compared with that under atmospheric CO_2_ concentration conditions; and (iii) to reveal the mechanism of split N applications on grain yield and quality of wheat from the perspective of N absorption, utilization, and distribution in wheat plants and grain protein fractions under e[CO_2_] conditions.

## 2 Materials and methods

### 2.1 Location

The experiment was conducted using the mini-FACE system of the Institute of Environment and Sustainable Development in Agriculture, Chinese Academy of Agricultural Sciences, from October 2020 to June 2022. The experimental site was located in Changping, Beijing (40°13′N, 116°14′E), China.

### 2.2 Agro-climatic conditions

**Figure 1:**
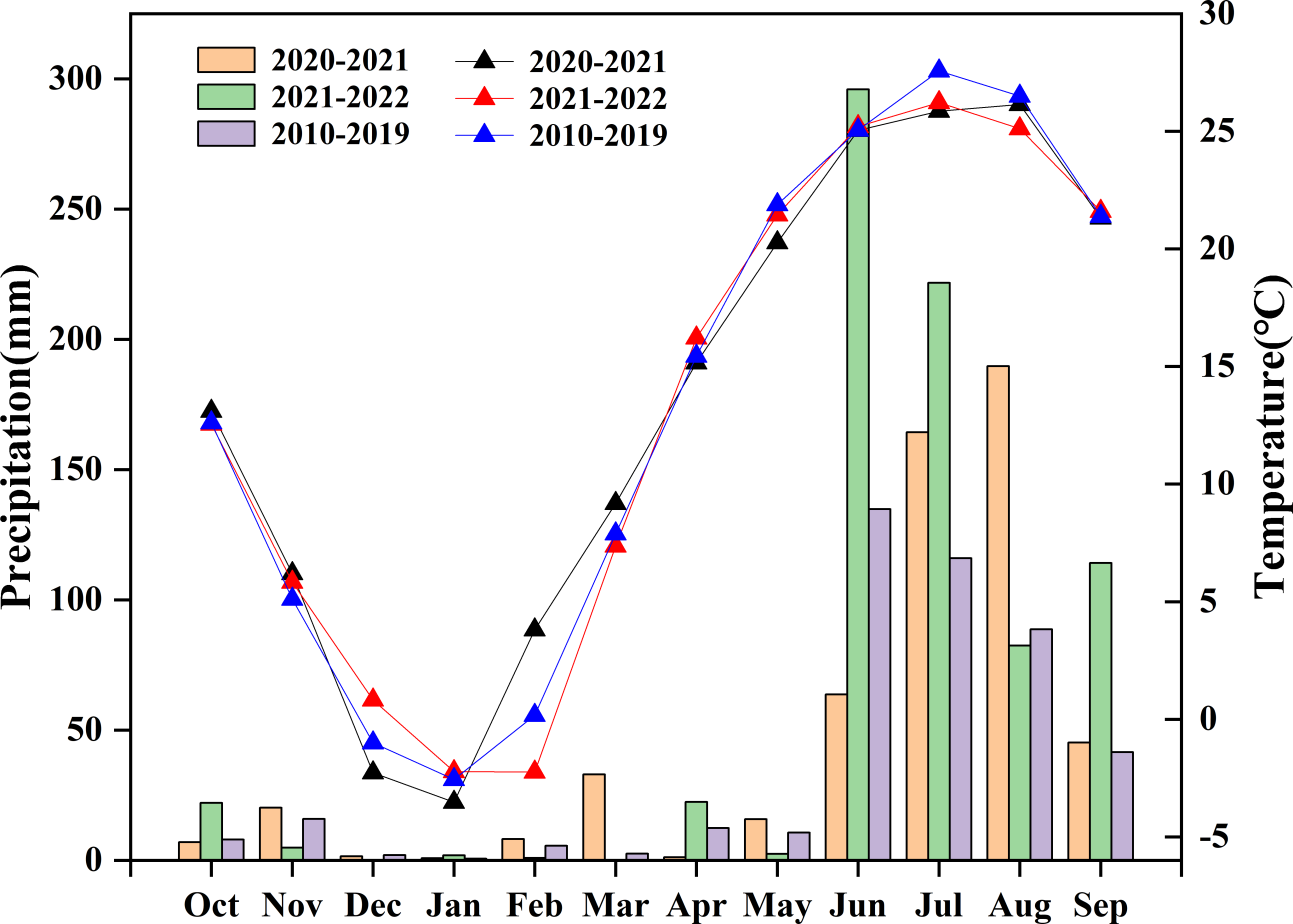
Monthly precipitation (bar) and temperature (line) during the experimental years in the growing period of wheat and mean values from 2010 to 2019 at the experimental site.

The region has a continental monsoon climate, with an average annual precipitation of 267 mm and an average annual temperature of 9.6°C. The average monthly precipitation and temperature for the test years 2010–2019 are summarized in **Figure 1**. The soil type (0–0.2 m) was a clay loam with a pH of 8.4, an organic matter content of 29.4 g·kg^-1^, total N content of 1.60 g·kg^-1^, available phosphorus content of 39.4 mg·kg^-1^, and available potassium content of 157.1 mg·kg^-1^.

### 2.3 Experimental procedures

Three FACE circles and three control circles were used for the experiment. Each FACE circle comprised eight CO_2_ gas release pipes with regular octagonal shapes, and the diameter of each circle was 4 m. The control circle was not installed with FACE pipes, and the other environmental conditions were consistent with the natural environmental conditions. The distance between each FACE and the control circle was greater than 14 m to eliminate interference from the CO_2_ concentrations. The increase in CO_2_ concentrations started at the beginning of March and ended in the middle of June of each year. The CO_2_ ventilation time was 6:30–18:30 every day.

### 2.4 Experimental design

This experiment used a split-plot design with two factors: CO_2_ concentrations and split N application timing. CO_2_ concentrations were the main treatment, with control and elevated CO_2_ concentrations. Control atmospheric CO_2_ (ACO_2_) was 400 ± 15 ppm, and elevated CO_2_ (ECO_2_) was 600 ± 15 ppm. The timing of the split N applications was a by-treatment, including no late stage N application (N_E_), split N application at the booting stage (N_B_), and split N application at the anthesis stage (N_F_). Six treatments were set up in the experiment, with three replicates (**Table 1**). Treatment 1 (AN_E_) and Treatment 4 (EN_E_) had 69 kg N hm^-2^ applied at the jointing stage, without split N applications at the late stage; Treatment 2 (AN_B_) and Treatment 5 (EN_B_) had 29 kg N hm^-2^ applied at the jointing stage and 40 kg N hm^-2^ at the booting stage; Treatment 3 (AN_F_) and Treatment 6 (EN_F_) had 29 kg N hm^-2^ applied at the jointing stage and 40 kg N hm^-2^ at the anthesis stage. The total N fertilizer application rate for each treatment was 212 kg N hm^-2^. Phosphate fertilizer (P_2_O_5_) and potassium fertilizer (K_2_O) were applied as basal fertilizers at 136 kg·hm^-2^ and 43 kg·hm^-2^, respectively. The base fertilizer was a mixed fertilizer, and the N fertilizer was urea (N ≥ 46%) in the late stage. Each experimental plot was 0.16 m^2^ (0.4 m × 0.4 m).

**Table 1:** Overview of CO_2_ level, N application rate, and timing in each treatment.

CO_2_ levels	Treatment	N application rate (kg N hm^-2^)			
		Before sowing	Stem elongation	Booting	Anthesis
ACO_2_	AN_E_	143	69		
	AN_B_	143	29	40	
	AN_F_	143	29		40
ECO_2_	EN_E_	143	69		
	EN_B_	143	29	40	
	EN_F_	143	29		40

Zhongmai 1062 winter wheat variety with high gluten content was used in this study. Wheat for the 2020–2021 season was sown on 6 October 2020, with a seeding rate of 187.5 kg·hm^-2^ and a row spacing of 0.2 m, and harvested on 19 June 2021. The 2021–2022 wheat season was sown on 21 October 2021, with a seeding rate of 225 kg·hm^-2^ and a row spacing of 0.2 m, and harvested on 17 June 2022. Other field management practices were conducted according to local standards.

### 2.5 Sample collection

At maturity, aboveground plant samples were collected, and 40 representative plants were selected to measure the grain yield and yield components. The remaining plants were separated into flag leaves, grains, glumes, cobs, remaining straws, and leaves. The fresh weight of samples was weighed and dried at 70°C to a constant weight.

### 2.6 Measurements

#### 2.6.1 Grain yield and yield components

A total of 40 plant samples were individually examined, and the number of ears and grains per plant was recorded and weighed. After drying, two groups of 500 grains each were randomly selected and weighed (accuracy set to 0.01 g). The 1000-grain weight was determined as follows: if the quotient of the difference between the two groups divided by the average value of the two groups did not exceed 5%, the sum of the weights of the two groups was used for the weight of 1000 grains; if the value was more than 5%, a third group was counted, and the two groups with the most similar weights were added to obtain the weight of 1000 grains.

#### 2.6.2 Nitrogen content, absorption, and utilization in aboveground wheat organs

The aboveground wheat organs were milled into powder using a universal high-speed grinding machine (FW100, Tianjin Taenite Instrument Co. Ltd., Tianjin, China), and the total N content of each organ was determined using the H_2_SO_4_-H_2_O_2_ digestion Kjeldahl method (Zhang et al., 2019). The values were then calculated using the following equations:


$$\text{N uptake of aboveground organs }\left({\text{g}\cdot \text{m}}^{-2}\right)\;=\text{organ biomass}\left({\text{g}\cdot \text{m}}^{-2}\right)\;\times \text{ total N content of each organ }\left(\%\right)$$

Total aboveground N uptake = sum of organ N uptake

$$\text{Harvest index }\left(\%\right)\;=\text{ grain yield }\left({\text{g}\cdot \text{m}}^{-2}\right)/\text{aboveground plant dry biomass }\left({\text{g}\cdot \text{m}}^{-2}\right)\;\times \;100$$

$$\text{N fertilizer partial factor productivity }\left({\text{g}\cdot \text{g}}^{-1}\right)\;=\text{ grain yield }\left({\text{g}\cdot \text{m}}^{-2}\right)/\text{N fertilizer application rate }\left({\text{g}\cdot \text{m}}^{-2}\right)$$

$$\text{N harvest index }\left(\%\right)\;=\text{ grain N uptake at maturity }\left({\text{g}\cdot \text{m}}^{-2}\right)/\text{aboveground plant N uptake }\left({\text{g}\cdot \text{m}}^{-2}\right)\;\times \;100$$

$$\text{N absorption efficiency }\left(\%\right)\;=\text{ plant N uptake }\left({\text{g}\cdot \text{m}}^{-2}\right)/\text{N fertilizer application rate}\left({\text{g}\cdot \text{m}}^{-2}\right)\;\times \;100$$

$$\text{N use efficiency }\left({\text{g}\cdot \text{g}}^{-1}\right)\;=\text{ grain yield }\left({\text{g}\cdot \text{m}}^{-2}\right)/\text{aboveground plant N uptake }\left({\text{g}\cdot \text{m}}^{-2}\right)$$

#### 2.6.3 Protein composition and content

Wheat grains were milled using a Laboratory Mill 120 (Perten, Sweden) to obtain whole wheat flour. Albumin, globulin, gliadin, and glutenin in the grains were sequentially extracted using a continuous extraction method as previously described (Zhang et al., 2019). The protein content was determined using the Kjeldahl method and calculated by multiplying the N content by 5.7 (Liu et al., 2005; Zhang et al., 2019).

### 2.7 Statistical analyses

The experiment was designed as a split-plot; the CO_2_ levels (atmospheric or elevated CO_2_) were the whole-plot treatment, and the split N application timing levels (jointing, booting, and anthesis stages) were the split-plot treatment. The ANOVAs were used to examine the effects of CO_2_ levels and split N application timing levels on yield performance (grain yield, ear number per unit area, grain number per ear, 1000-grain weight, and harvest index), quality traits (protein, albumin, globulin, gliadin, glutenin, and gluten), and N use traits (grain and aboveground N uptake, N uptake efficiency, partial factor productivity of N fertilizer, and N harvest index) using the statistical software R 4.2.3. The LSD was performed at the 5% probability level in ANOVAs. The general linear mixed model was performed to determine the effect of the year. A normal distribution test was performed for each model. The CO_2_ level and split N application timing level were considered fixed factors, and the year, CO_2_ concentrations, and block were considered random factors (Mitchell, 2003) when comparing the differences in yield performance, quality traits, and N use traits among different treatments in different years. If they did not follow a normal distribution, the data were converted to meet a normal distribution using different methods (Zuur et al., 2009). Origin Pro 2023 was used as the graphing software.

## 3 Results

### 3.1 Grain yield and components

**Figure 2:**
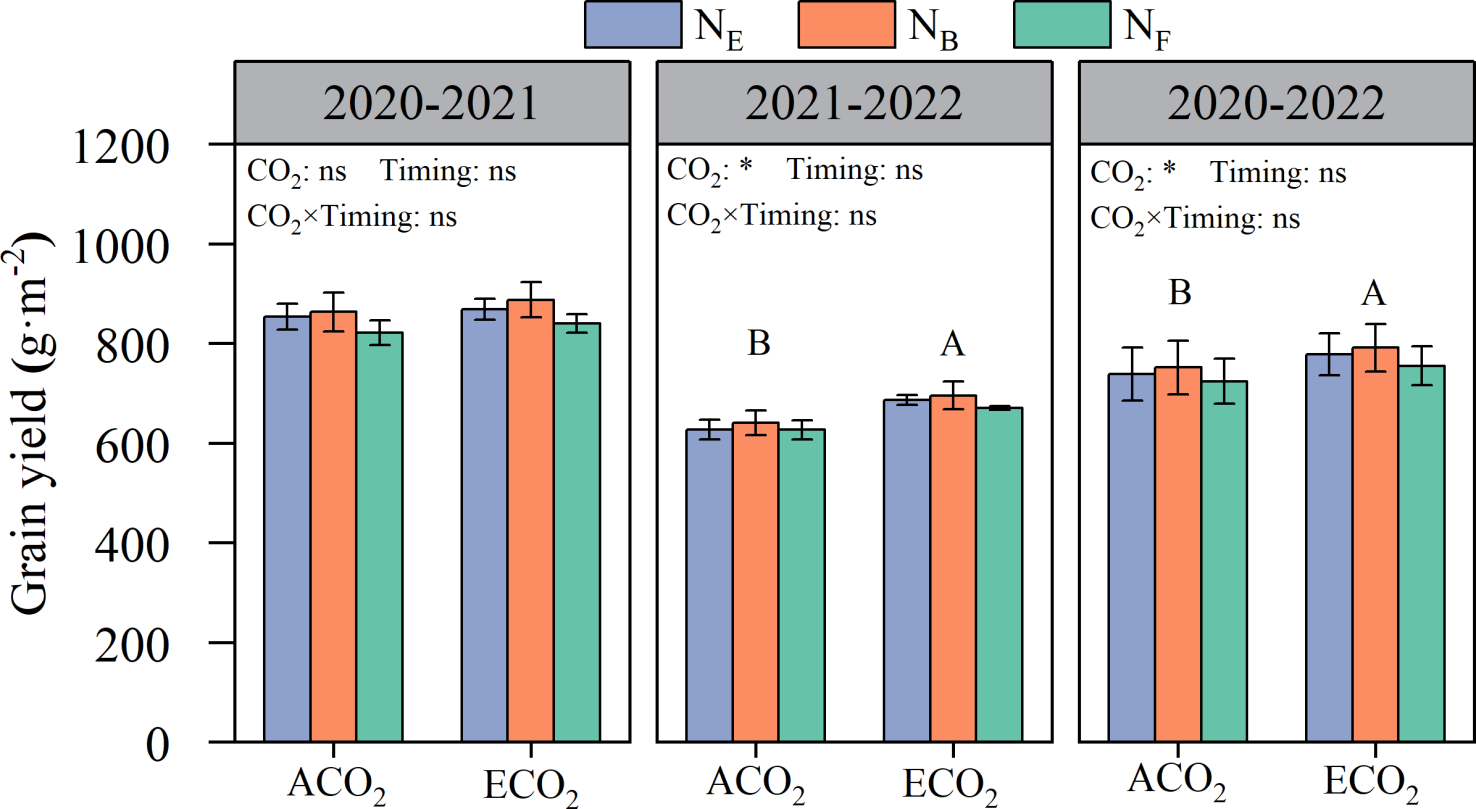
Effects of e[CO_2_] and split N application on wheat grain yield. Error bars represent the standard error. Multiple comparisons were performed within the same year: lowercase letters indicate significant differences between treatments at different N application periods at the same CO_2_ concentrations in the same year (*p*< 0.05); uppercase letters indicate significant differences between treatments at different CO_2_ concentrations in the same year (*p* < 0.05). ns, not significant; *p< 0. 05.

Elevated atmospheric CO_2_ concentrations were beneficial in increasing wheat grain yield, but there were significant differences between the years (**Figure 2**). Wheat grain yields in 2020–2021 and 2021–2022 amounted to 821.0–887.2 g·m^-2^ and 626.3–694.9 g·m^-2^, respectively. Compared to ACO_2_, ECO_2_ increased wheat yield by an average of 5.0% in both seasons. Wheat grain yield was not influenced by split N applications under ACO_2_ and ECO_2_ conditions.

According to the analysis of yield components and harvest index of wheat in both seasons (**Table 2**), CO_2_ concentrations and split N applications at the late growth stage had no significant effect on the number of ears per unit area of wheat. However, ECO_2_ significantly increased the number of grains per spike, 1000-grain weight, and harvest index of wheat by 3.03, 1.98, and 1.58%, respectively, compared to ACO_2_. The split N application at the late growth stages significantly affected the 1000-grain weight and harvest index of wheat. Compared to no split N application (EN_E_), the split N application under ECO_2_ conditions increased the 1000-grain weight by 1.12% at the booting stage (EN_B_) and decreased it by 1.12% at anthesis (EN_F_). No significant effects were observed under the ACO_2_ conditions. In addition, under ACO_2_ and ECO_2_ conditions, the wheat harvest index with split N application at the booting stage was the highest and increased by 2.52% and 1.56%, respectively, compared to that without split N application. However, this effect was not observed in split N applications at anthesis.

**Table 2:** Effects of elevated atmospheric CO_2_ concentrations and N topdressing timing on wheat yield components and harvest index.

Year	CO_2_ levels	Treatment	Number of ears per unit area (m^-2^)	Grains per ear	1000-grain weight (g)	Harvest index (%)
2020-2021	ACO_2_	N_E_	595.8 ± 20.5 aA	29.8 ± 0.3 aA	47.4 ± 0.1 aA	42.3 ± 0.3 bA
		N_B_	602.1 ± 17.1 aA	28.2 ± 0.8 aA	47.8 ± 0.1 aA	43.8 ± 0.2 aA
		N_F_	597.9 ± 18.5 aA	28.8 ± 0.8 aA	47.4 ± 0.4 aA	42.1 ± 0.3 bA
	ECO_2_	N_E_	580.8 ± 18.4 aA	29.4 ± 0.9 aA	47.7 ± 0.3 abA	42.8 ± 0.5 aA
		N_B_	600.0 ± 18.8 aA	29.2 ± 0.5 aA	48.5 ± 0.2 aA	43.8 ± 0.3 aA
		N_F_	573.8 ± 14.2 aA	30.0 ± 0.2 aA	47.3 ± 0.2 bA	42.2 ± 0.5 aA
2021-2022	ACO_2_	N_E_	500.0 ± 9.5 aA	27.6 ± 0.6 aA	40.4 ± 0.3 aB	45.4 ± 0.2 bB
		N_B_	494.2 ± 16.9 aA	28.9 ± 0.6 aA	39.9 ± 0.2 aB	46.2 ± 0.2 aA
		N_F_	497.9 ± 18.2 aA	28.3 ± 0.9 aA	39.9 ± 0.1 aB	45.3 ± 0.2 bB
	ECO_2_	N_E_	555.0 ± 16.8 aA	30.0 ± 0.6 aA	41.8 ± 0.2 aA	46.8 ± 0.3 aA
		N_B_	541.7 ± 24.0 aA	29.3 ± 0.7 aA	41.9 ± 0.1 aA	47.2 ± 0.5 aA
		N_F_	535.4 ± 11.0 aA	28.9 ± 0.7 aA	41.2 ± 0.1 bA	46.7 ± 0.2 aA
2020-2022	ACO_2_	N_E_	547.9 ± 23.7 aA	28.7 ± 0.6 aA	43.9 ± 1.6 aB	43.9 ± 0.7 bB
		N_B_	548.1 ± 26.4 aA	28.5 ± 0.5 aA	43.9 ± 1.8 aB	45.0 ± 0.6 aA
		N_F_	547.9 ± 25.2 aA	28.6 ± 0.6 aA	43.7 ± 1.7 aA	43.7 ± 0.8 bA
	ECO_2_	N_E_	567.9 ± 12.6 aA	29.7 ± 0.5 aA	44.7 ± 1.3 bA	44.8 ± 1.0 bA
		N_B_	570.8 ± 18.9 aA	29.3 ± 0.4 aA	45.2 ± 1.5 aA	45.5 ± 0.8 aA
		N_F_	554.6 ± 11.8 aA	29.4 ± 0.4 aA	44.2 ± 1.4 cA	44.4 ± 1.0 bA
ANOVA results	Year		***	ns	***	***
	CO_2_		ns	*	***	***
	Timing		ns	ns	**	***
	Year×CO_2_		**	ns	***	*
	Year×Timing		ns	ns	*	ns
	CO_2_×Timing		ns	ns	ns	ns
	Year×CO_2_×Timing		ns	ns	ns	ns

Error bars represent the standard error. Multiple comparisons were performed within the same year: lowercase letters indicate significant differences between treatments at different N application rates at the same CO_2_ concentrations in the same year (p< 0.05); uppercase letters indicate significant differences between treatments at different CO_2_ concentrations in the same year (p< 0.05). ns, not significant; *p< 0.05; **p< 0.01; ***p< 0.001.

### 3.2 Grain protein concentration and protein composition

**Figure 3:**
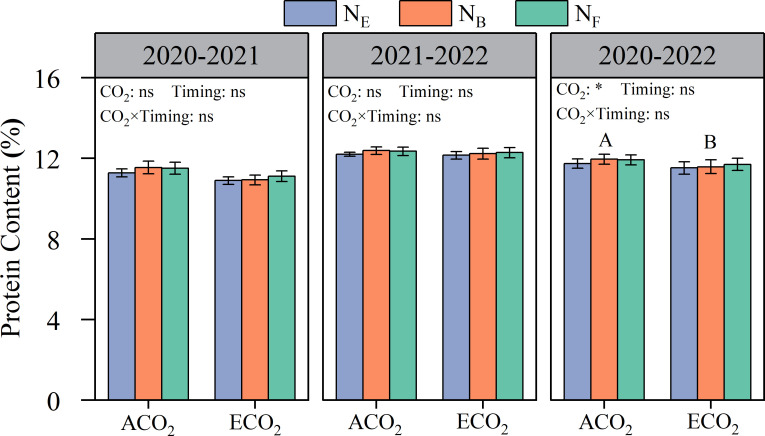
Effects of e[CO_2_] and split N application on grain protein concentration in wheat grain. Error bars represent the standard error. Multiple comparisons were performed within the same year: lowercase letters indicate significant differences between treatments at different N application rates at the same CO_2_ concentrations in the same year (*p*< 0.05); uppercase letters indicate significant differences between treatments at different CO_2_ concentrations in the same year (*p*< 0.05). ns, not significant; *p< 0.05.

Elevated atmospheric CO_2_ concentrations were generally detrimental to wheat grain protein content, but there were significant differences between the years (**Figure 3**). The grain protein content of wheat in 2020–2021 and 2021–2022 was 10.9–11.5% and 12.2–12.4%, respectively. On average, the grain protein content of wheat under ECO_2_ conditions was reduced by 2.32% compared to that under ACO_2_ conditions. There were no significant effects of the different late stage split N applications on wheat grain protein content under both ACO_2_ and ECO_2_ conditions.

**Figure 4:**
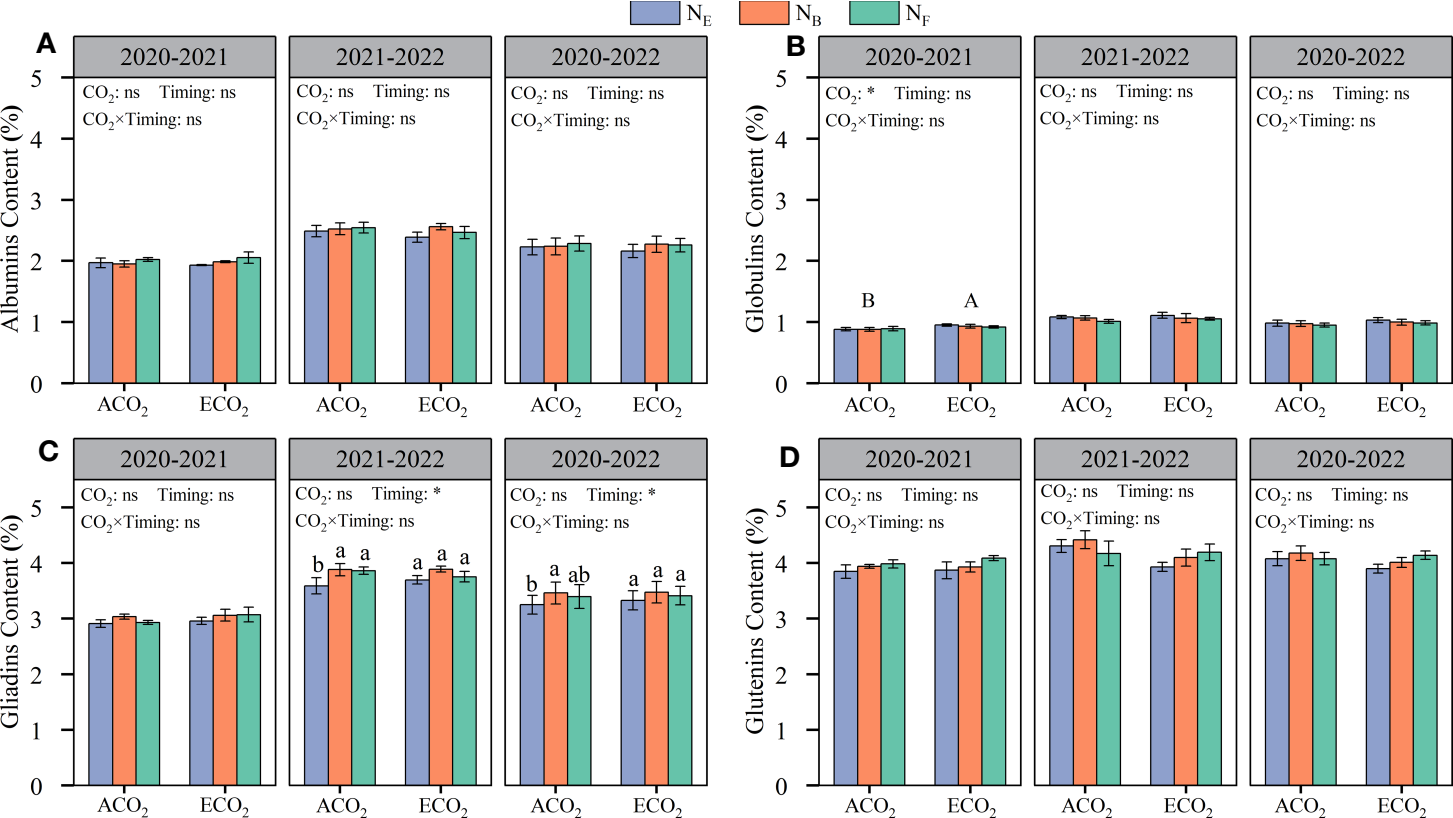
Effects of e[CO2] and split N application on albumins content **(A)**, globulims content **(B)**, gliadims content **(C)**, and glutenins content **(D)** in wheat grain. Error bars represent the standard error. Multiple comparisons were performed within the same year: lowercase letters indicate significant differences between treatments at different N application rates at the same CO2 concentrations in the same year (p < 0.05); uppercase letters indicate significant differences between treatments at different CO2 concentrations in the same year (p < 0.05). ns, not significant; *p < 0.05.

From the two-year average value, CO_2_ concentrations had no significant effect on the grain protein composition (albumin, globulin, gliadin, and glutenin contents) of wheat (**Figure 4**). The wheat grain contained 2.16–2.33% albumin, 0.95–1.03% globulin, 3.25–3.48% gliadin, and 3.90–4.18% glutenin. The gliadin content of wheat grains was significantly affected by late stage split N application; under ACO_2_ conditions, split N application at the booting stage (AN_B_) increased gliadin content by 6.43% compared to that without split N application (AN_E_) (**Figure 4C**).

**Figure 5:**
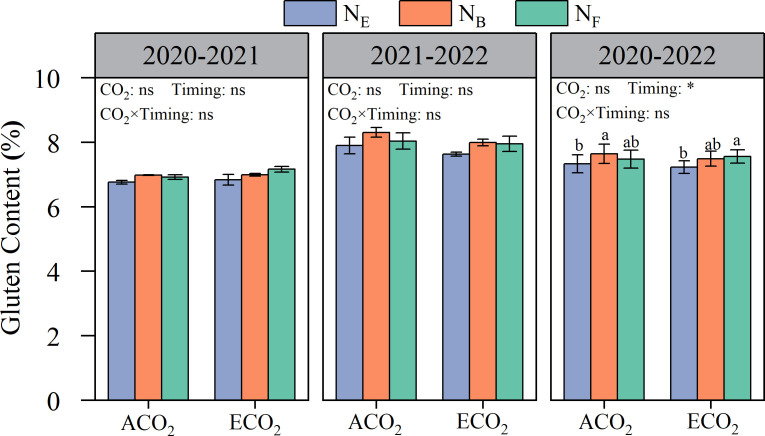
Effects of e[CO_2_] and split N application on the gluten protein content of wheat grain. Error bars represent the standard error. Multiple comparisons were performed within the same year: lowercase letters indicate significant differences between treatments at different N application rates at the same CO_2_ concentrations in the same year (*p*< 0.05); uppercase letters indicate significant differences between treatments at different CO_2_ concentrations in the same year (*p*< 0.05). ns, not significant; *p< 0.05.

Although the effects on the content of gliadin and glutenin in wheat grains were inconsistent, different split N applications at the late stages had significant effects on the content of gluten (gliadin and glutenin) in wheat grains (**Figure 5**). Under ACO_2_ and ECO_2_ conditions, late stage split N application increased gluten protein content by 3.12% and 4.06%, respectively, compared to that without split N application. Under ACO_2_ conditions, AN_B_ was more effective than AN_E_ in increasing grain gluten protein by 4.24%. In contrast, under ECO_2_ conditions, EN_F_ was more effective than EN_E_ in increasing grain gluten protein by 4.52%. The results showed that the key timing of split N application for improving grain gluten protein was delayed under ECO_2_ conditions compared to that under ACO_2_ conditions.

### 3.3 N uptake and utilization

**Figure 6:**
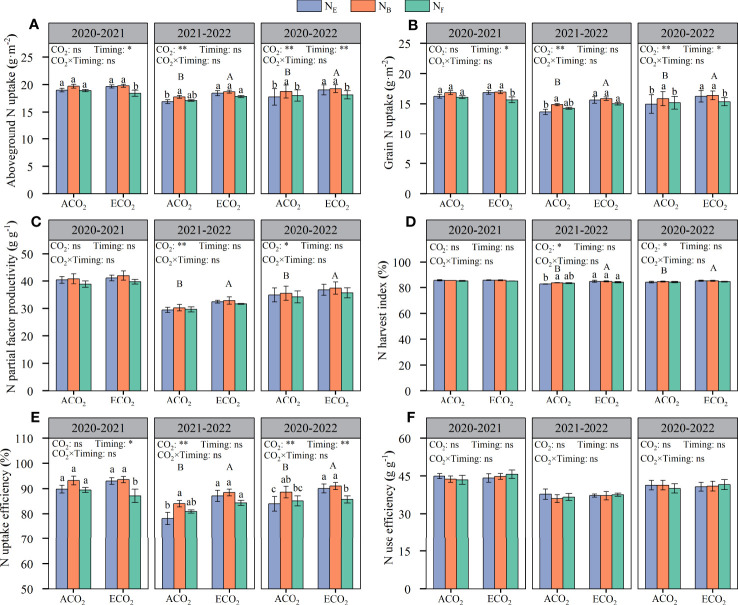
Effects of e[CO2] and split N application on aboveground N uptake **(A)**, grain N uptake **(B)**, N partial factor productivity **(C)**, N harvest index **(D)**, N uptake efficiency **(E)**, and N use efficiency **(F)** in wheat. Error bars represent the standard error. Multiple comparisons were performed within the same year: lowercase letters indicate significant differences between treatments at different N application rates at the same CO2 concentrations in the same year (p < 0.05); uppercase letters indicate significant differences between treatments at different CO2 concentrations in the same year (p < 0.05). ns, not significant; *p < 0.05; **p < 0.01.

The elevated atmospheric CO_2_ concentrations significantly increased the aboveground N uptake of wheat, but there were significant differences between years (**Figure 6A**). The aboveground N uptake of wheat in 2020–2021 and 2021–2022 amounted to 18.42–19.78 g·m^-2^ and 16.91–18.70 g·m^-2^, respectively. The aboveground N uptake of wheat in both seasons increased by 3.21% under ECO_2_ conditions compared to that under ACO_2_ conditions. The aboveground N uptake of wheat was significantly affected by the split N application at the late growth stage. AN_B_ increased by 4.55% compared to AN_E_ under ACO_2_ conditions, whereas there was no such effect with split N applications at anthesis (AN_F_). Under ECO_2_ conditions, there was no difference between EN_B_ and EN_E_, while EN_F_ reduced aboveground N uptake of wheat by 4.82% compared to EN_E_.

From the two-year average value, the grain N uptake for each treatment was analyzed with the same significance as that of the aboveground N uptake (**Figures 6A, B**). The grain N uptake of wheat in 2020–2021 and 2021–2022 amounted to 15.66–16.95 g·m^-2^ and 13.67–15.86 g·m^-2^, respectively. The N uptake of wheat grains significantly increased by 4.34% under ECO_2_ conditions compared to that under ACO_2_ conditions. In addition, the N uptake of wheat grains was significantly affected by late stage split N applications (**Table 3**). Under ACO_2_ and ECO_2_ conditions, the N uptake of wheat grains with split N applications at the booting stage was the highest, increasing by 5.98% and 0.98%, respectively, compared to that without split N application, whereas there was no such effect with split N applications at anthesis. The results showed that split N applications at the booting stage promoted the uptake and accumulation of N in wheat and grains.

**Table 3:** Results of the ANOVA analysis.

		Grain yield (g·m^-2^)	Protein Content (%)	Albumins Content (%)	Globulins Content (%)	Gliadins Content (%)	Glutenins Content (%)	Gluten Content (%)	Grain N uptake (g·m^-2^)	Aboveground N uptake (g·m^-2^)	N harvest index (%)	N uptake efficiency (%)	N use efficiency (g·g^-1^)	N partial factor productivity (g·g^-1^)
ANOVA results	Year	**	**	**	**	**	*	**	**	**	**	**	**	**
	CO_2_	*	*	ns	ns	ns	ns	ns	**	**	*	**	ns	*
	Timing	ns	ns	ns	ns	*	ns	*	*	**	ns	ns	ns	ns
	Year×CO_2_	ns	ns	ns	ns	ns	ns	ns	**	*	*	*	ns	ns
	Year×Timing	ns	ns	ns	ns	ns	ns	ns	ns	ns	ns	ns	ns	ns
	CO_2_×Timing	ns	ns	ns	ns	ns	ns	ns	ns	ns	ns	*	ns	ns
	Year×CO_2_×Timing	ns	ns	ns	ns	ns	ns	ns	ns	ns	ns	ns	ns	ns

Error bars represent the standard error. ns, not significant; *p< 0.05; **p< 0.01; ***p< 0.001.

The elevated atmospheric CO_2_ concentrations were beneficial for increasing the partial factor productivity of N fertilizers and the N harvest index, but there were significant differences between the years (**Figures 6C, D**). Under ECO_2_, the partial factor productivity of N fertilizers and harvest index increased by 4.96% and 1.15%, respectively, compared to those under ACO_2_. Under ACO_2_ and ECO_2_ conditions, there was no significant effect of split N application on the partial factor productivity of N fertilizers or the N harvest index. Although there was a significant effect of ECO_2_ and split N application on the N uptake efficiency of wheat (**Figure 6E**), there was no significant interaction effect on the N use efficiency of wheat (**Figure 6F**). The results showed that elevated atmospheric CO_2_ concentrations increased grain yield and promoted N uptake in wheat. The N use efficiency of wheat grain in 2020–2021 and 2021–2022 was 43.46–45.65% and 36.03–37.79%, respectively. Overall, grain N uptake was not affected by CO_2_ concentrations.

## 4 Discussion

### 4.1 Yield components influenced by e[CO_2_] and split N application in wheat

High variations in wheat grain yield were achieved between seasons; yield ranged from 821.0 to 887.2 g·m^-2^ in 2020–2021 and from 626.3 to 694.9 g·m^-2^ in 2021–2022. The relatively low grain yield in the 2021–2022 season may have resulted from high precipitation in the wheat grain-filling period, which impacted the growth of wheat grain, leading to a much lower kernel weight than that in 2020–2021 (**Figure 1** and **Table 2**). Wheat grain yield was increased (5.0%) by ECO_2_ compared to that by ACO_2_ in this study (**Figure 2**), which is consistent with several studies reporting that e[CO_2_] can improve wheat grain yield (Wang et al., 2013; Han et al., 2015; Dier et al., 2018; Dier et al., 2019). e[CO_2_] improves the net photosynthetic rate of wheat leaves by promoting the carboxylation reaction of RuBisCO to improve the carboxylation rate and reducing photorespiration by inhibiting the oxidative competition of RuBisCO enzymes, thus enhancing the net photosynthetic efficiency. An increase in photosynthetic efficiency is conducive to photosynthetic carbon assimilation, thereby increasing carbohydrate accumulation (Hasan et al., 2021; Hamani et al., 2023). Although the highest grain yield was obtained when late N applications were split at the booting stage, no significant effects of split N application on grain yield were observed in either the ACO_2_ or ECO_2_ treatments. One possible reason is that the N supply from fertilizer and soil was sufficient to meet the N demand of wheat, which ensured the tillering and growth of wheat because the total N and organic matter contents in the soil were at medium to high levels (Navarro et al., 2020). On the other hand, the N loss from the soil decreased due to inhibition in the nitrifying and denitrifying communities, which ensured N availability for wheat (Liu et al., 2023). Further analysis of the yield components and harvest index showed that the number of kernels per ear, 1000-grain weight, and harvest index of wheat increased under e[CO_2_] conditions. Moreover, split N applications significantly influenced the 1000-kernel weight and harvest index of wheat (**Table 2**). These results indicate that e[CO_2_] during grain filling limits the carbon sink but enriches the N sink capacity, as demonstrated by other studies, thus increasing the N demand of wheat (Martínez-Peña et al., 2022; Martínez-Peña et al., 2023). Split N applications at the late growth stages (booting or anthesis) met the elevated N demand of wheat during grain filling due to e[CO_2_], which prolonged the grain-filling period (Kong et al., 2016) and promoted dry matter accumulation and transport to the grains, leading to an improved grain weight and harvest index of wheat.

### 4.2 Grain protein composition influenced by e[CO_2_] and split N applications in wheat

Consistent with most studies (Högy et al., 2013; Walker et al., 2017; Rezvi et al., 2023), the results of the present study clearly show that e[CO_2_] decreased GPC (**Figure 3**) because of the higher rate of increase in grain yield (5.0%, **Figure 2**) compared to that of grain N uptake (3.2%, **Figure 6**), resulting in a dilution effect in GPC. In addition, other mechanisms such as lowered transpiration (reduced nutrient acquisition in the roots due to reduced mass flow in the soil and diminished nutrient translocation *via* the xylem sap in the shoots), disruption of N assimilation (reduced photorespiration leads to reduced $\text{N}{\text{O}}_{3}^{-}$ assimilation and N content in the shoots due to the insufficient NADH in the cytosol to drive the $\text{N}{\text{O}}_{3}^{-}$ reduction reaction), and regulation of root N uptake and signaling (deregulated $\text{N}{\text{O}}_{3}^{-}$ uptake systems in the root leading to a lower rate of $\text{N}{\text{O}}_{3}^{-}$ acquisition) may also exert a great contribution to the decrease in GPC under e[CO_2_] conditions (Gojon et al., 2023). e[CO_2_] increased carbon sources, and limited storage capacity led to excessive accumulation of soluble sugars, such as sucrose and starch, in photosynthetic organs, resulting in an imbalance in the source-sink relationship of carbon and N in wheat (Hasan et al., 2021). Furthermore, although the GPC was reduced, the grain protein composition (albumins, globulins, gliadins, and glutenins) was not altered by e[CO_2_] (**Figures 4** and **5**). This is inconsistent with Wieser et al. (2008), who reported that gliadin and glutenin content decreased by 20% and 15%, respectively, under e[CO_2_] conditions. Besides, Soba et al. (2019) found that e[CO_2_] increased gluten content by 32% and gliadin content by 79.6%, while glutenin content decreased by 4.3%. It is concluded that e[CO_2_] altered the N metabolism and the kinetics of protein accumulation, suggesting a critical impact on amino acid synthesis, N remobilization, and redistribution (Singh et al., 2020). However, the grain protein composition was altered by split N applications. Gluten protein content (gliadins and glutenins) was increased by split N applications at booting and anthesis under the ACO_2_ and ECO_2_ treatments, respectively, compared to that without split N applications (**Figure 5**). Changes in gliadin content were more sensitive to split N applications than those in glutenin content, which may be closely related to the synthesis of grain protein fractions (Rossmann et al., 2019; Xue et al., 2019). Structural proteins (mainly albumins and globulins) begin to accumulate in the early stages of wheat grain development, are synthesized and accumulated from anthesis to approximately 20 days post-anthesis, and are mainly regulated by sinks (Martre et al., 2003). However, gluten proteins (mainly gliadins and glutenins, accounting for approximately 80% of grain protein) are mainly considered source-regulated and begin to synthesize and accumulate from around 7 to 10 days post-anthesis until the end of grain filling, with gliadins being synthesized slightly earlier than glutenins (Barneix, 2007). Therefore, since N absorbed post-anthesis contributes more directly to grain N accumulation (Blandino et al., 2015), split N applications at late growth stages of wheat may induce the reprogramming of various biosynthetic pathways, such as hormone signal transduction and photosynthesis, by regulating leaf senescence and N reabsorption to provide more available N for grains, thus promoting gluten (especially gliadin) synthesis based on meeting N demand for structural protein synthesis (Zhong et al., 2023). In addition, the key timing of split N applications for optimizing grain protein composition differed under atmospheric CO_2_ and e[CO_2_] conditions. Compared to that without split N applications, the gluten protein content of wheat grains improved when late N was applied at booting (AN_B_) and anthesis (EN_F_) under atmospheric CO_2_ (ACO_2_) and e[CO_2_] (ECO_2_) conditions, respectively (**Figure 5**). One possible reason for this is that e[CO_2_] promotes dry matter and N accumulation in wheat (**Figure 6**) and increases N translocation and its contribution to grain N accumulation compared to atmospheric CO_2_ concentration conditions (Högy et al., 2009; Dier et al., 2019). Therefore, split N applications at a relatively later stage (at anthesis compared to the booting stage) provided a more effective N source for gluten synthesis and accumulation and altered N distribution in grain protein fractions (Hellemans et al., 2018; Rossmann et al., 2019; Ar et al., 2020).

In conclusion, e[CO_2_] increased the grain yield while reducing GPC in wheat. Although split N applications at the late growth stages of wheat could not eliminate the negative effect of e[CO_2_] on GPC, grain protein composition was improved owing to the optimized distribution of N into different grain protein fractions, leading to an increase in gluten protein content, which was beneficial for wheat quality improvement. In addition, the key timing for split N applications in coordinating the yield and quality of wheat by improving grain protein composition was postponed from the booting stage to around anthesis under e[CO_2_] conditions compared to that under atmospheric CO_2_ concentration conditions. Therefore, it is possible to achieve both high wheat yield and quality without increasing the input of N fertilizer through the rational application of N fertilizer at the critical growth stage of wheat under elevated atmospheric CO_2_ concentrations.

## 5 Conclusion

e[CO_2_] can increase the grain yield but reduce the protein content of wheat. The application of N fertilizer (split from the N applied at the jointing stage) at the late growth stage of wheat did not alleviate the negative effects of e[CO_2_]. However, it can alter the distribution of N in the grain protein fractions to increase the gluten protein content, which is conducive to improving the processing quality of wheat. The key timing for split N applications in coordinating wheat yield and quality by improving grain protein composition should be moved from the booting stage to around anthesis under e[CO_2_] conditions. Results from the present study indicate that rational handling of N fertilizer applications at the critical growth stage may be a promising approach to coordinate grain yield and quality of wheat under future climate change conditions. This offers the potential to maintain or even reduce N fertilizer inputs when N fertilizers are rationally distributed during wheat growth at the optimal time for wheat production under elevated atmospheric CO_2_ concentrations in the future.

## Data availability statement

The original contributions presented in the study are included in the article/supplementary material. Further inquiries can be directed to the corresponding author.

## Author contributions

YP conducted the experiments under the supervision of CX. YP and XH performed the statistical analysis and wrote the manuscript. CX revised the manuscript. XH, HX, WW, XL, and YL finalized the experiments and the manuscript. All authors contributed to the article and approved the submitted version.
